# Supplementary material for: Restriction-modification systems are required for Neisseria gonorrhoeae pilin antigenic variation
Source: bioRxiv. 2026 Jan 14:2026.01.10.698803. Preprint. [Version 1] doi: 10.64898/2026.01.10.698803 (PMC12871193; doi:10.64898/2026.01.10.698803)
Supplement: Supplement 1 [file NIHPP2026.01.10.698803v1-supplement-1.pdf]

bioRxiv preprint doi: https://doi.org/10.64898/2026.01.10.698803; this version posted January 14, 2026. The copyright holder for this preprint (which was not certified by peer review) is the author/funder, who has granted bioRxiv a license to display the preprint in perpetuity. It is made available under aCC-BY 4.0 International license.

EXTENDED DATA

Table S1. List of the predicted Type II RM with their predicted recognition motif and the number of occurrences in the Gc strain FA1090 N-1-60.

Predicted Type II RM system	Motif (5' to 3')	Number of occurrences in FA1090 N-1-60	Number of non-overlapping occurrences in FA1090 N-1-60
**NgoFVII**	GCSGC	16173	13952
**NgoAXIV and NgoAXIII**	CCGG	11103	7884
**NgoAII**	GGCC	4590	2018
**NgoAXIP**	GATC	2259	2112
**NgoAXV**	GGNNCC	2033	818
**NgoAX**	CCACC	1575	1380
**NgoAIV**	GCCGGC	1572	0
**NgoAI**	RGCGCY	622	224
**NgoAVIII**	GACNNNNNTGA	407	338
**NgoAIII**	CCGCGG	218	45
**NgoAXVII**	GAGNNNNNTAC	118	89

Table S2 – Number of occurrences of each of the known motifs targeted by type II restriction-modification systems in Gc strain FA1090

Motif (5' to 3')	# in *pilE*	# in 19 *pilS* copies
GCSGC	2	41
CCGG	4	87
GGCC	3	48
GATC	1	1
GGNNCC	0	14
CCACC	1	12
GCCGGC	1	32
RGCGCY	0	8
GACNNNNNTGA	0	1
CCGCGG	0	0
GAGNNNNNTAC	0	0

bioRxiv preprint doi: https://doi.org/10.64898/2026.01.10.698803; this version posted January 14, 2026. The copyright holder for this preprint (which was not certified by peer review) is the author/funder, who has granted bioRxiv a license to display the preprint in perpetuity. It is made available under aCC-BY 4.0 International license.

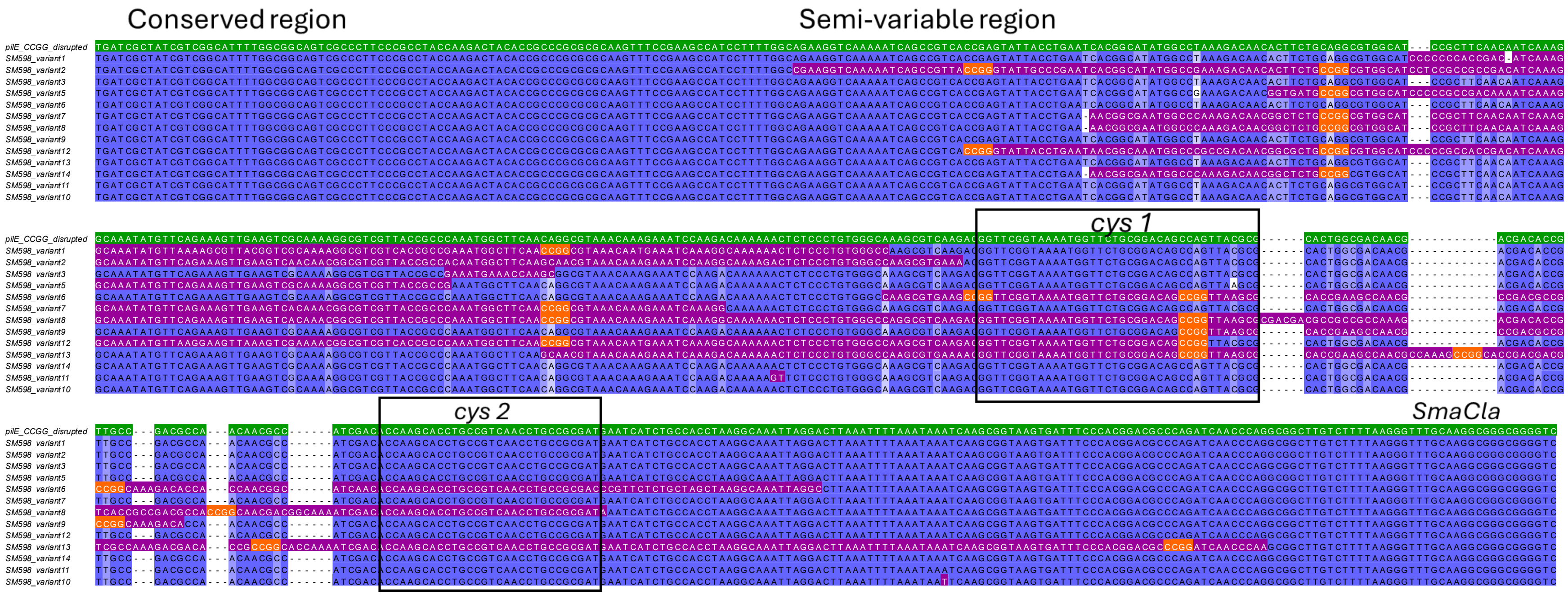

Fig. S1- Sequence alignment of *pilE* amplicon from the SM598 mutant variants. The variants were reisolated from blebs on GCB IPTG after 30h of growth. The reference sequence is indicated in green, the *pilS* inserts are indicated in purple and the 5'-CCGG sites are indicated in orange.

bioRxiv preprint doi: https://doi.org/10.64898/2026.01.10.698803; this version posted January 14, 2026. The copyright holder for this preprint (which was not certified by peer review) is the author/funder, who has granted bioRxiv a license to display the preprint in perpetuity. It is made available under aCC-BY 4.0 International license.

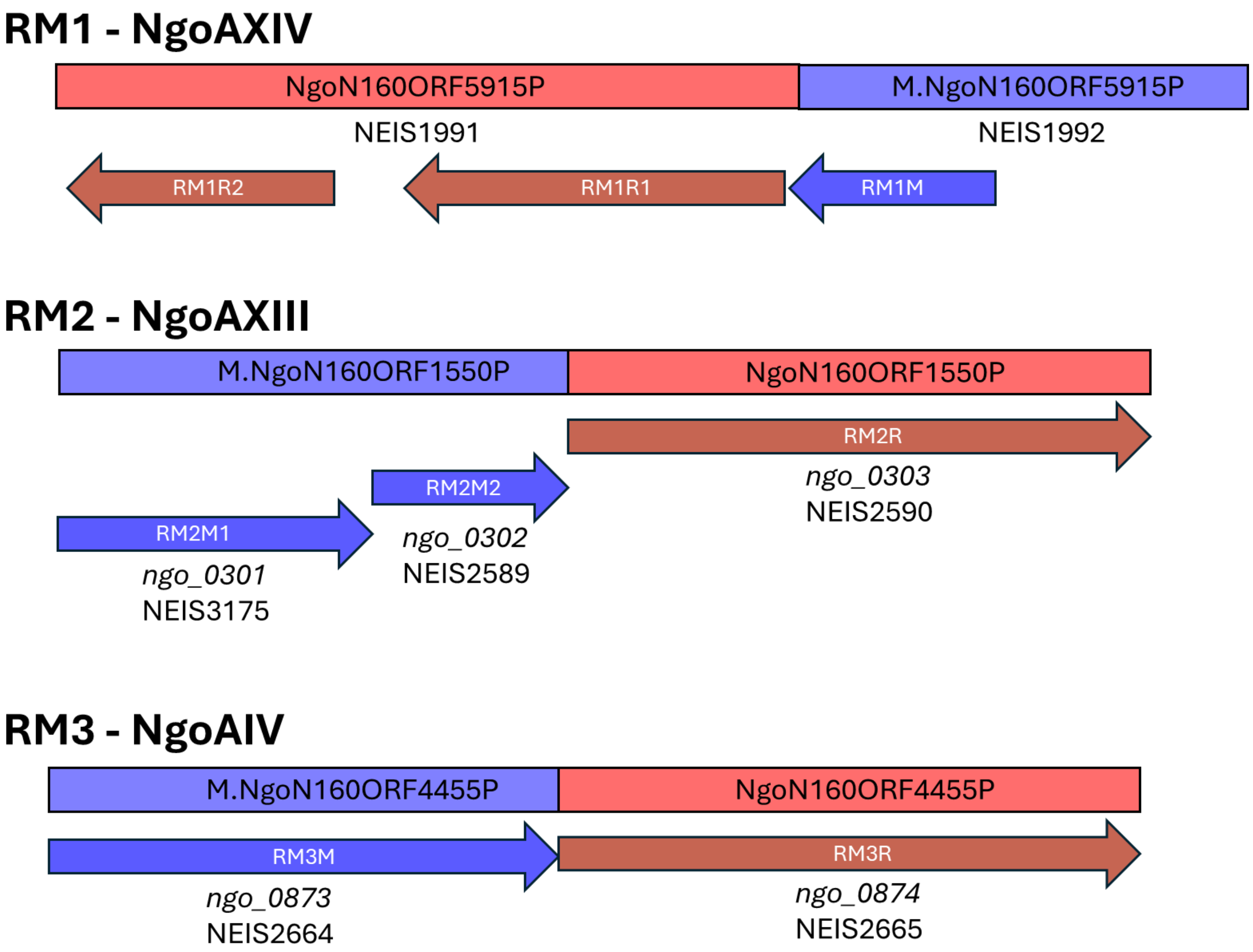

Fig. S2- Genomic organization of the RM1 RM2 and RM3 operons in Gc FA1090. Rectangles follow Rebase nomenclature. ORFs are labeled with Ngo_ identifiers, while NEIS numbers corresponds to PubMLST nomenclature. Red indicates restriction genes; blue, methylase genes.

bioRxiv preprint doi: https://doi.org/10.64898/2026.01.10.698803; this version posted January 14, 2026. The copyright holder for this preprint (which was not certified by peer review) is the author/funder, who has granted bioRxiv a license to display the preprint in perpetuity. It is made available under aCC-BY 4.0 International license.

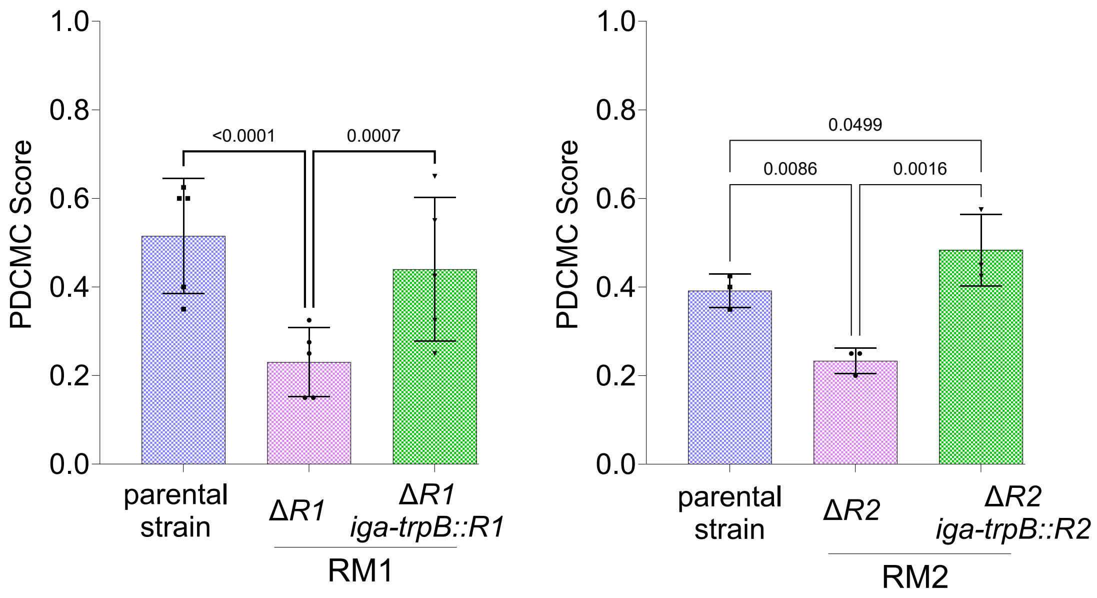

Fig. S3 – PDCMC score measured in the strains Δ*R1.RM1* (SM323) and Δ*R.RM2* (SM327) and their complements. The gene of interest RMR1 on the left, RM2R on the right was reinserted in the *iga-trpB* site using pMR69. Fischer's LSD test was used, only the p-values <0.05 were plotted.

bioRxiv preprint doi: https://doi.org/10.64898/2026.01.10.698803; this version posted January 14, 2026. The copyright holder for this preprint (which was not certified by peer review) is the author/funder, who has granted bioRxiv a license to display the preprint in perpetuity. It is made available under aCC-BY 4.0 International license.

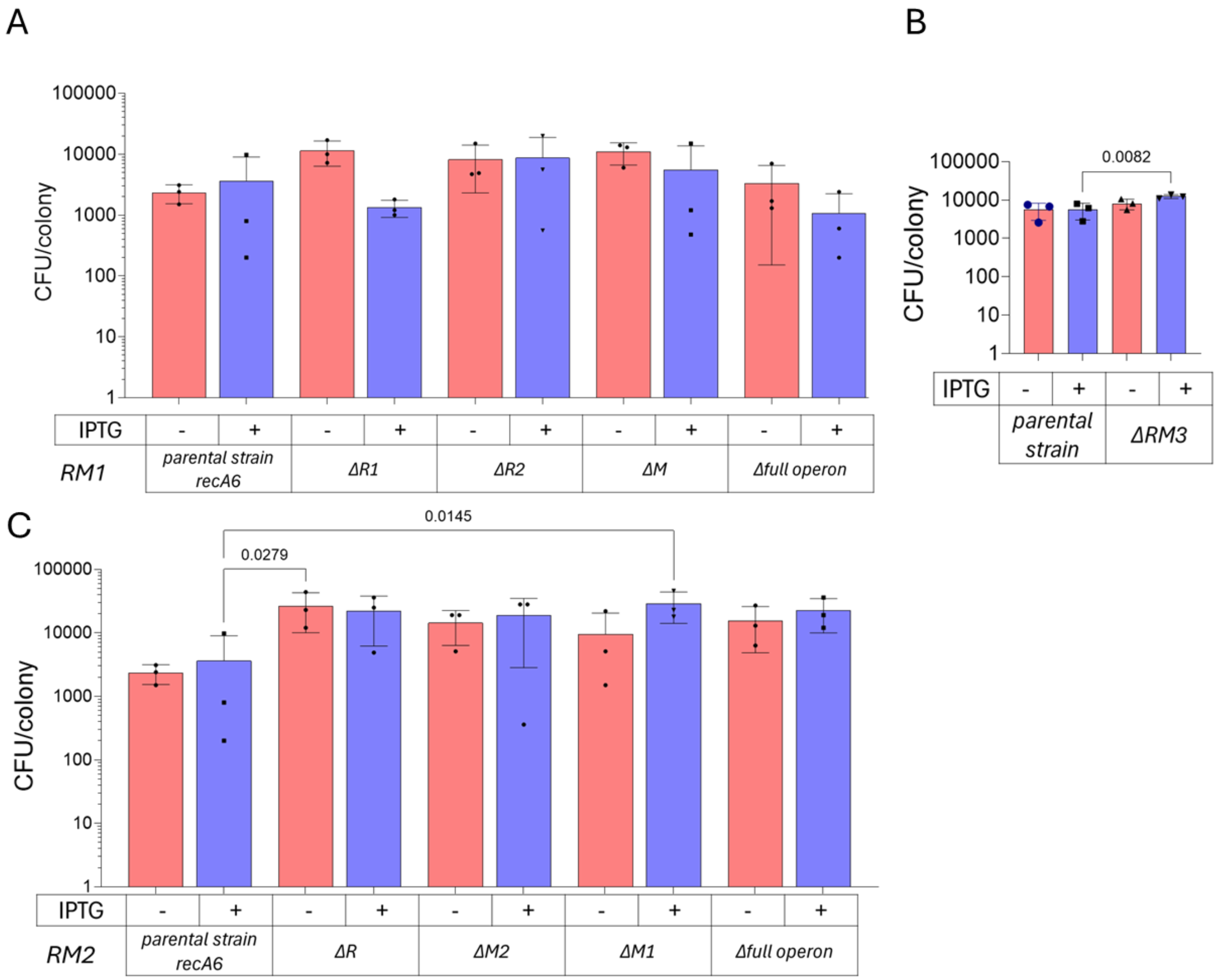

Fig. S4 – Measure of the growth of mutants carrying single gene deletions of the three operons. CFU/colony of the strains of the individual mutants for the operons **A.** RM1, **B.** RM3, and **C**. RM2, after 30h of growth on GCB with and without IPTG. Multiple comparisons were performed using Fischer's LSD test, only the p-values <0.05 were plotted.

bioRxiv preprint doi: https://doi.org/10.64898/2026.01.10.698803; this version posted January 14, 2026. The copyright holder for this preprint (which was not certified by peer review) is the author/funder, who has granted bioRxiv a license to display the preprint in perpetuity. It is made available under aCC-BY 4.0 International license.

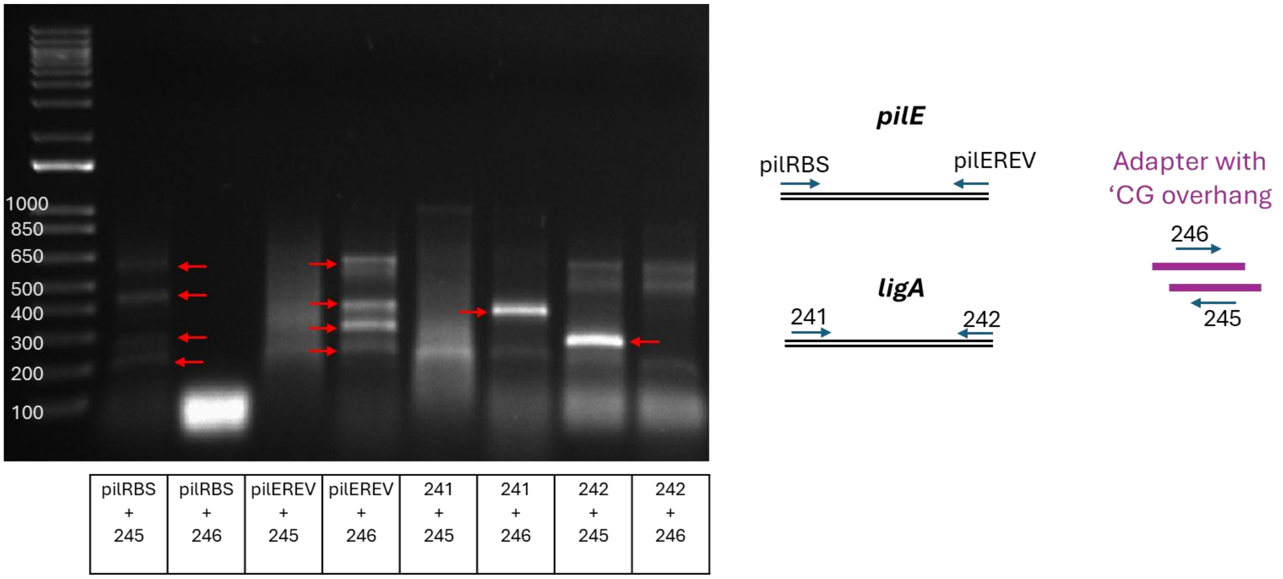

Fig. S5 – Ligation of the adapter within the genomic DNA. Migration of the PCR-amplified fragments using ligated genomic DNA as a template and several pairs of primers where one of them is internal to the ligated adapter (246 or 245). The bands expected if the adapter has ligated into a 5'-CCGG cut are boxed in reindicated with red arrows. The *ligA* gene is used as a control fragment that contains only one 5'-CCGG site.

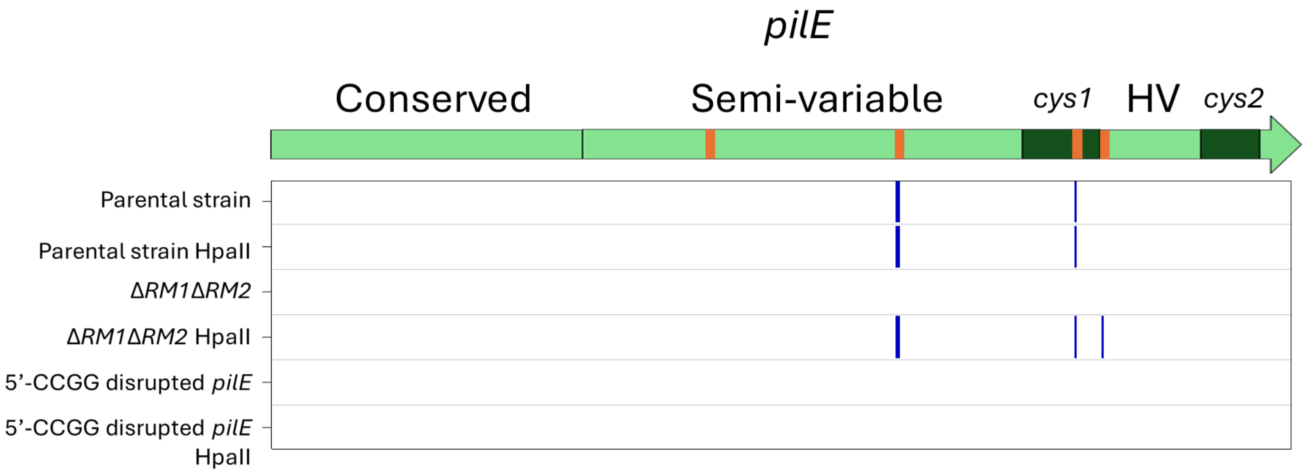

Fig. S6. Detecting CCGG cuts within the *pilE* gene. Detection of the adapter within *pilE*. The schematic representation of the *pilE* shows its different regions with the 5'-CCGG sites are represented in orange. The panel below the *pilE* gene shows the locations of the detected CCGG cuts in, from top to bottom: the parental strain *recA6*, it's HpaII-pretreated control, followed with the RM1RM2 double mutant (SM500), its HpaII control, the CCGG-disrupted strain (SM598) and its HpaII control.

bioRxiv preprint doi: https://doi.org/10.64898/2026.01.10.698803; this version posted January 14, 2026. The copyright holder for this preprint (which was not certified by peer review) is the author/funder, who has granted bioRxiv a license to display the preprint in perpetuity. It is made available under aCC-BY 4.0 International license.

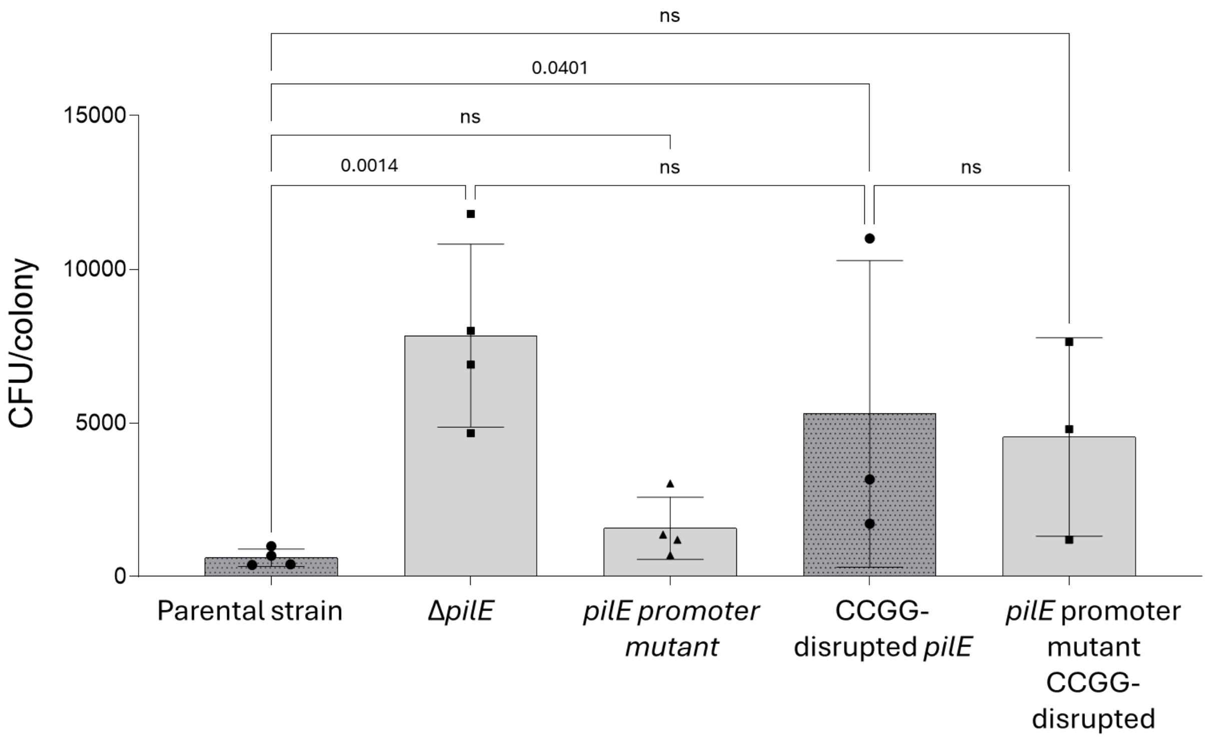

Fig. S7. Measure of the growth of mutants carrying *pilE* mutations. CFU/colony of the strains Δ*pilE*, *pilE* promoter mutant, *pilE* CCGG-disrupted mutant and a mutant bearing both mutations at 16h of growth on GCB with IPTGMultiple comparisons were performed using Fischer's LSD test.

Table S3. Strains used in this study

**Bacterial strains**		
FA1090 *recA6*	[62]	*recA6*
FA1090 *recA6* CCGG disrupted *pilE*	This study	SM598
FA1090 *recA6* Δ*R1.RM1*	This study	SM323
FA1090 *recA6* Δ*R2.RM1*	This study	SM325
FA1090 *recA6* Δ*M.RM1*	This study	SM356
FA1090 *recA6* Δ*RM1*	This study	SM310
FA1090 *recA6* Δ*M1.RM2*	This study	SM362
FA1090 *recA6* Δ*M2.RM2*	This study	SM360
FA1090 *recA6* Δ*R.RM2*	This study	SM327
FA1090 *recA6* Δ*RM2*	This study	SM364

bioRxiv preprint doi: https://doi.org/10.64898/2026.01.10.698803; this version posted January 14, 2026. The copyright holder for this preprint (which was not certified by peer review) is the author/funder, who has granted bioRxiv a license to display the preprint in perpetuity. It is made available under aCC-BY 4.0 International license.

FA1090 *recA6 ΔRM1ΔRM2*	This study	SM500
FA1090 *recA6 ΔR.RM3*	This study	SM600
N-1-60 CRISPRi ngo0873 (M.RM3)	This study	SM602
FA1090 *recA6 ΔRM3*	This study	SM604
FA1090 *recA6 Δ*$pilS_{123678}$ hexadeleted mutant	Shaohui Yin	Q409
FA1090 *recA6 Δ*$pilS_{123678}$ hexadeleted mutant KanR	This study	SM564
FA1090 *recA6* hexadeleted mutant::*pilS3C1* KanR	This study	SM568
FA1090 *recA6* hexadeleted mutant::CCGG-disrupted *pilS3C1* KanR	This study	SM570
FA1090 *recA6* hexadeleted mutant *pilE* CCGG-disrupted KanR	This study	SM582
FA1090 *recA6* hexadeleted mutant::*pilS3C1 pilE* CCGG-disrupted KanR	This study	SM584
FA1090 *recA6* hexadeleted mutant::CCGG-disrupted *pilS3C1* CCGG-disrupted *pilE* KanR	This study	SM585
FA1090 *recA6 ΔR1.RM1 R1.RM1* at *iga-trpB*	This study	SM527
FA1090 *recA6 ΔR.RM2 R.RM2* at *iga-trpB*	This study	SM560
FA1090 *recA6 ΔpilE*	[63]	SM648
FA1090 *recA6 pilE*-10::NheI	[47]	SM650
FA1090 *recA6* CCGG-disrupted *pilE*-10::NheI	This study	SM655
